# Small Size, No versus Limited Fluoroscopy, and Other Challenges of Pediatric Ablation Procedures

**DOI:** 10.19102/icrm.2019.100605

**Published:** 2019-06-15

**Authors:** Kathryn K. Collins

**Affiliations:** ^1^Division of Pediatric Cardiology, Children’s Hospital Colorado, Aurora, CO, USA

**Keywords:** Catheter ablation, fluoroscopy, pediatric, supraventricular tachycardia

Catheter ablation for the management of arrhythmia substrates has been available as a treatment option for almost 30 years. Over that time, significant technological efforts have been made in electroanatomic systems to decrease fluoroscopy exposure, improve mapping of the arrhythmia substrate, and refine the delivery of ablative energy (be it cryoablative energy or radiofrequency energy). In the current issue of the *Journal of Innovations in Cardiac Rhythm Management*, Dr. Chang^1^ presents a complex case study of an infant who underwent catheter ablation for ectopic atrial tachycardia in a manner that serves to highlight some of the challenges that persist regarding conducting ablations in the very young. In this supplementary commentary, I would like to highlight three key aspects that must be carefully kept in mind in pediatric patients undergoing such procedures, as follows: patient size, the use of no versus limited fluoroscopy, and maintaining limited vascular access.

With respect to patient size, the indications for catheter ablation in pediatrics have changed over time. In 2002, a consensus statement on ablation in pediatric patients from the then-called North American Society for Pacing and Electrophysiology dictated a IIA-level recommendation for catheter ablation in children with a normal heart structure and good ventricular function with recurrent and/or symptomatic supraventricular tachycardia (SVT) refractory to conventional therapy who were older than four years of age.^2^ In 2016, as part of the newest recommendations for ablation in pediatrics from the Heart Rhythm Society, ablation in a similar patient population was made a class I recommendation.^3^ There have also been modifications made to the language, defining “larger patients” to be those heavier than 15 kg rather than basing the classification on an age criteria as previously was true. Another modification made was to the stipulation of the SVT needing to be refractory to medical therapy, in that the current recommendations for ablation have been broadened to include if “the family wishes to avoid [the use of] chronic antiarrhythmic medications.” In practice, these recommendations have translated to ablation being a reasonable therapeutic option in patients with normal heart structure and function who are heavier than 15 kg and who have documented SVT without the need to control the tachycardia with medications prior to ablation. Families are given the option of clinically monitoring with no therapy, medical management, or invasive electrophysiology study and ablation. Anecdotally, in my clinical practice, I have a very few number of families who opt to pursue medical management of their child’s SVT; as ablation procedures are highly efficacious and are associated with low recurrences and low complication risks, I instead find that most families pursue a cure.

However, what about the smaller children and infants weighing less than 15 kg? Existing recommendations state that ablation can be useful in this population but that “medical therapy should be considered” prior to ablation.^3^ Indeed, in the case presented by Dr. Chang,^1^ the infant had failed multiple medications including a combination of medications prior to the author proceeding to ablation. The decision of when medical management has failed is very operator- and institution-dependent—as well it should be, given that the comfort of the operator in conducting infant ablation is a necessity to achieve better procedural outcomes. Initial publications of ablation procedures in the young have reported higher major complication rates in patients weighing less than 15 kg including for significant complications such as myocardial infarction or pericardial effusion.^4^ The more recent literature, however, has drawn conclusions that show excellent success rates in infants and with the main complication being vascular access issues.^5^ Importantly, as part of a search of PubMed, I could find no report of a patient death following infant catheter ablation for SVT in the recent era. The one caveat here could be publication bias, in that successful outcomes and procedures are more likely to be submitted for publication versus unsuccessful ones. One of my mentors often would say that “medicine makes one humble,” and I often think of this phrase when I conduct an infant catheter ablation. As operators, we have had the luxury of high success and low complication rates. It only takes one serious complication, however, to shift one’s thinking about any invasive or surgical option for our patients. As the indication for catheter ablation is put forth for younger and smaller patients, I would advocate for a level of humility and caution in making decisions as to when a very small infant should undergo a catheter ablation procedure.

Concerning the use of fluoroscopy in catheter ablations, much has been written on decreasing fluoroscopy exposure during invasive electrophysiology procedures and ablations. There remains a debate ongoing as to “no” versus “limited” fluoroscopy exposure being the ideal goal for procedures. Every aspect of the procedural approach is evolving: when I started my career, fluoroscopy was always utilized to check wire position during vascular access. Now, ultrasound is more widely employed for this task. Additionally, electroanatomic mapping systems allow for visualization of catheter movement without the use of fluoroscopy. These systems are widely accepted as a necessary piece of equipment in an ablation laboratory. Echocardiography (transthoracic, transesophageal, or intracardiac ultrasound) has also been put forth as an alternate technology for visualization during transseptal puncture procedures. In one recent publication on fluoroscopy use in a pediatric center, approximately 80% of their ablation cases were conducted with less than one minute of fluoroscopy time.^6^ This constitutes a significant improvement from typical practices even 10 years ago. It is clear that the field is moving toward, although the widespread achievement and implementation of zero-fluoroscopy procedures remain to be attained.

As Dr. Chang^1^ discusses, all of these technologies have challenges surrounding their application in very small infants. Modifications to patches are required to successfully place all monitoring and safety equipment on the very limited skin surface area of infants. There are also limitations in the size of the echocardiogram or ultrasound probes. Every operator and institution needs to pursue the use of technology in their laboratory while considering both the risks and benefits of the use of that technology at their specific facility.

I would focus briefly on the statement by Dr. Chang that “fluoroscopy was required during vascular access and catheter advancement.” It is my experience that comfort with nonfluoroscopic techniques has to do with how one was trained and how often an operator utilizes the nonfluoroscopic techniques. The “requirement” of fluoroscopy is true at the discretion of the operator. One example is the placement of a catheter in the coronary sinus (CS). When operators in our laboratory began using nonfluoroscopic systems for catheter placement in the CS, fluoroscopy was often utilized when the catheter would not advance easily. Eventually, though, with constant use of the system and dedication to learning the limits of the technique, all operators now place the CS catheter without fluoroscopy and in fact do not wear protective lead aprons for most ablation procedures. The most junior operator has never placed a CS catheter with fluoroscopy and finds fluoroscopy images not helpful in this portion of the procedure. Thus, my assessment for the requirement of fluoroscopy is based on operator comfort with the benefits and limitations of the available technology. Each operator should strive to pursue the system that has the highest chance for success and lowest risk of complications to the patient.

Finally, I would like to consider vascular access and catheter choice in small infants. I am in agreement with Dr. Chang’s statement that a “less is better” strategy is often utilized in younger patients.^1^ Alternatives such as esophageal catheters for left-sided atrial electrograms or as reference catheters are often considered. A report by Ozaki et al. found that fewer catheters were utilized in patients aged one year to four years old versus those aged between five years and nine years in their patient population.^7^ In many articles addressing complications in younger patients, vascular access concerns are often the highest complication risk (although not the most major of complications). In our laboratory, a “standard” invasive electrophysiology procedure involves only three sites of intravenous access for the placement of a high right atrial catheter, a CS catheter, and a combined HIS and right ventricular catheter, respectively. (This is decreased from a prior standard protocol of four catheters including separate HIS and right ventricular catheters.) The use of fewer catheters does not appear to alter the efficacy of the ablation procedure.

In summary, catheter ablation for arrhythmia substrates in the pediatric population are highly efficacious with a low complication rate. The technologic advancements in ablation procedures achieved for larger patients can also be applied in smaller patients, but their usage may require preprocedural planning in terms of numbers and types of catheters inserted as well as considerations made regarding what modifications are required to accommodate the patient’s size.
